# Simulating the Evolution of Functional Brain Networks in Alzheimer’s Disease: Exploring Disease Dynamics from the Perspective of Global Activity

**DOI:** 10.1038/srep34156

**Published:** 2016-09-28

**Authors:** Wei Li, Miao Wang, Wenzhen Zhu, Yuanyuan Qin, Yue Huang, Xi Chen

**Affiliations:** 1 School of Automation, Huazhong University of Science and Technology, Wuhan, 430074, P. R. China; 2 Image Processing and Intelligent Control Key Laboratory of Education Ministry of China, Wuhan, 430074, P. R. China; 3 China Ship Development and Design Center, Wuhan, 430064, P. R. China; 4 Department of Radiology, Tongji Hospital, Tongji Medical College, Huazhong University of Science and Technology, Wuhan, 430074, P. R. China; 5 School of Electrical and Electronic Engineering, East China Jiaotong University, Nanchang, 330013, P. R. China

## Abstract

Functional brain connectivity is altered during the pathological processes of Alzheimer’s disease (AD), but the specific evolutional rules are insufficiently understood. Resting-state functional magnetic resonance imaging indicates that the functional brain networks of individuals with AD tend to be disrupted in hub-like nodes, shifting from a small world architecture to a random profile. Here, we proposed a novel evolution model based on computational experiments to simulate the transition of functional brain networks from normal to AD. Specifically, we simulated the rearrangement of edges in a pathological process by a high probability of disconnecting edges between hub-like nodes, and by generating edges between random pair of nodes. Subsequently, four topological properties and a nodal distribution were used to evaluate our model. Compared with random evolution as a null model, our model captured well the topological alteration of functional brain networks during the pathological process. Moreover, we implemented two kinds of network attack to imitate the damage incurred by the brain in AD. Topological changes were better explained by ‘hub attacks’ than by ‘random attacks’, indicating the fragility of hubs in individuals with AD. This model clarifies the disruption of functional brain networks in AD, providing a new perspective on topological alterations.

Alzheimer’s disease is the leading cause of dementia^1^, accounting for approximately 50–80% of all dementia cases^2^. AD is a progressive brain disorder. Previous studies have observed orderly atrophy in several cortical areas in different disease stages^3^,^4^, possibly characterising an underlying rearrangement of brain connectivity after neural injury. Due to its non-invasive nature and convenient process of data acquisition, resting-state functional magnetic resonance imaging (rs-fMRI) has been widely used to investigate the pathological changes *in vivo*^5^.

Graph theory is an effective tool for studying the topological organization of complex networks^6^. It has also been widely utilized to measure the topological changes in functional brain networks^7^,^8^. In its theoretical framework, the brain is described as a graph consisting of nodes and edges. Specifically, nodes represent regions or voxels in the brain, and edges represent the connections or couplings between nodes. A brain can therefore be characterised by topological properties that measure information transfer performance or failure tolerances. A healthy brain network is usually reported to have a higher clustering coefficient and a shorter characteristic path length than a random one, exhibiting small-world architecture characterised by an optimal balance between local segregation and global integration^9^,^10^,^11^. However, the clustering coefficient is reported to be significantly lower in the networks of individuals with AD, resulting in a disruption of the small-world property^12^,^13^,^14^,^15^. Sanz-Arigita *et al.*^15^ found a decrease in characteristic path length in AD, showing values approaching the theoretical values of random networks. Healthy brain networks are therefore considered to shift from a small-world architecture to a random profile during the pathological process. In a graph, hubs are defined as highly connected nodes, mediating an economical trade-off between topological value and biological cost^16^. Due to their central role and high cost, hubs are suggested to be highly vulnerable in AD individuals. Specifically, cortical hubs concentrate most of the amyloid-β deposition in AD^17^,^18^. Thus, AD is likely to preferentially impact brain network hubs^19^.

Using graph theory, we can systematically investigate the inherent differences in topological properties between the brain networks of individuals with AD and those of normal controls (NC). From this static perspective, alterations in brain connectivity can help us to characterise the brain disorder. However, the pathological process is progressive and continuous. Dynamic and static factors coexist in the formation of brain disease. Thus, a deterministic connectome pattern cannot fully describe the alterations that accompany pathological processes in functional brain networks: A wealth of information is omitted regarding intermediate processes in the evolution from healthy to diseased brain networks. We are particularly interested in how alterations in the arrangement of connections in functional brain networks can lead to differences in topological properties. In this study, we proposed a dynamic model to simulate the pathological processes of AD, based on computational experiments. In a previous study, Vertes *et al.*^20^ proposed generative models that ‘produce’ a functional brain network from scratch, starting with an isolated node; normal brain-like networks can be generated using their approach. However, this growth model increased connections in networks but did not implement synaptic pruning or cell death. On the other hand, there were inherent differences between the pathological process and normal aging, as a result, specific procedures should be considered in abnormal evolution. Stam *et al.*^19^ proposed a failure model of functional brain networks in AD, in which failures always occur on edges connecting hubs, resulting in a transition from the NC to AD brain. Strictly speaking, this is not an evolution model, because no new edges grow or reconnect in the networks. It is more realistic that in a real brain additional regions would be recruited to compensate for functional deficits^21^,^22^.

In the present study, we first investigated the difference in several topological properties between the NC and individuals with AD. Then, we proposed an evolutionary model, to simulate the rearrangement of connections during the pathological process, starting from the NC group, and ending with the characteristics of the AD group. More specifically, we hypothesised that, during the pathological process of AD, edge disconnections in functional brain networks would tend to occur between hub-like nodes to disrupt the small-world architecture, and edge connections were more likely to occur between two random nodes, to generate a randomised profile. Subsequently, we evaluated our model by examining whether the topological properties of the simulated networks after evolution were consistent with those of the real AD group. Finally, we further examined whether our specific procedures used to form the evolution model were successful by two network-attack experiments.

## Methods

### Participants

In our study, the participants were recruited from two subject pools. The first was the Alzheimer’s Disease Neuroimaging Initiative (ADNI) database (http://adni.loni.usc.edu/), including 22 AD individuals and 22 healthy normal controls. Only baseline scans were utilized in order to avoid using multiple scans of the same subject. The second set of participants were recruited from Tongji Hospital. Recruitment was approved by the internal Institutional Review Board of Tongji Hospital. The methods were carried out in accordance with the approved guidelines, including any relevant details. Informed consent was obtained from all participants.

We randomly selected 15 AD and 15 NC samples from the ADNI database as the primary cohort for the main experiments. The remaining subjects in the ADNI database were used as an independent cohort for validation of our conclusions. The subjects recruited from Tongji Hospital constituted another independent cohort for verification purposes as we considered that only one independent cohort from the same subject pool would be underpowered to validate the experimental results. The demographic data of the three cohorts are shown in Table 1.

### Data acquisition and preprocessing

All scans in the primary cohort were acquired on a 3.0T Philips scanner with the following parameters: TR/TE = 3000/30, FA = 80°, slice thickness = 3.3 mm, matrix = 64 × 64, number of slices = 48, time points = 140. For more information regarding the data acquisition, please see http://www.adni-info.org.

The preprocessing of raw scans was carried out using Statistical Parametric Mapping (SPM12, http://www.fil.ion.ucl.ac.uk/spm) and Data Processing Assistant for Resting-State fMRI (DPARSF)^23^. The first ten images of each subject were discarded to ensure magnetisation equilibrium. Then, we corrected all of the data in the time domain by slice timing. Subsequently, all images were realigned to remove movement artefacts. Subjects whose head translation exceeded 2 mm or whose head rotation exceeded 2° were excluded (all participants shown in Table 1 passed these criteria). All images were normalized to the MNI template for consistency. The images were subsequently spatially smoothed with a standard 4 × 4 × 4 FWHM kernel. The time courses were filtered with a band-pass frequency range from 0.01 Hz to 0.08 Hz, to preserve low-frequency fluctuations^24^,^25^. Finally, the covariates, consisting of six head motion parameters, the global mean signal, white matter signal, and cerebrospinal fluid signal, were removed.

### Construction of functional brain network

**Figure 1:**
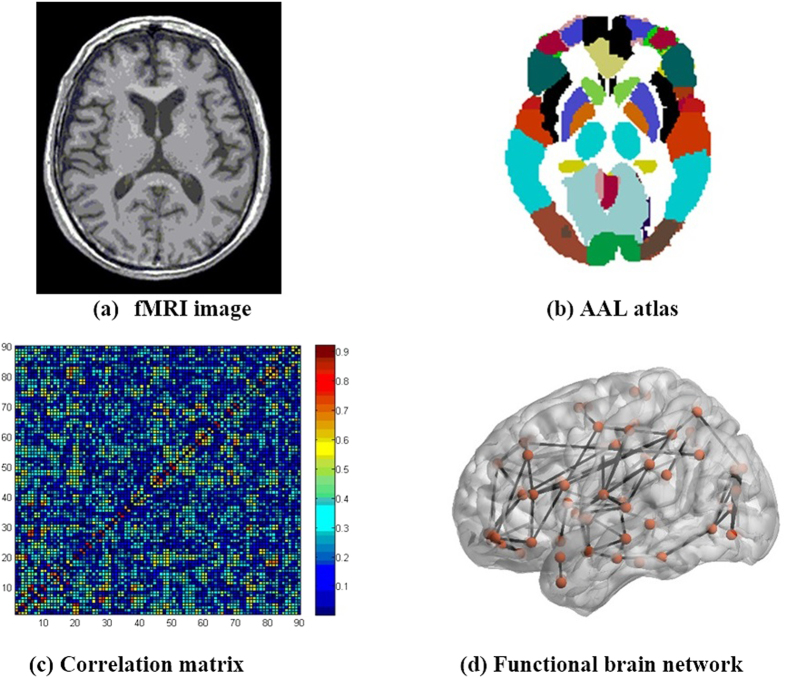
The process of constructing a functional brain network. (**a**) The brain images were parcelled into 90 ROIs, which were defined by (**b**) the automatic anatomical labelling (AAL) atlas. (**c**) An individual-specific correlation matrix was obtained by calculating the absolute correlation coefficients between each pair of ROIs. (**d**) By thresholding, the individual functional brain connectivity was obtained.

For each individual, the brain images (Fig. 1a) were parcelled into 90 ROIs by automatic anatomical labelling (AAL)^26^,^27^,^28^, as shown in Fig. 1b (the names of the ROIs are provided in the Supplementary materials). Time courses within each region were subsequently extracted. The absolute Pearson correlation coefficients between each pair of different time courses were calculated to represent the strength of corresponding connections. Consequently, each individual’s specific correlation matrix was obtained (Fig. 1c). The diagonal elements are zeros because they had no meaning in this context. We then fixed the matrix to be binary symmetric by restricting the network density. Taking a density of 30% for example, we set the top 30% of coefficients in the correlation matrix to 1, and the remaining coefficients to 0. To explore our model over a variety of conditions, we constructed the networks in a large density range, from 22 to 52%. The reasons for this density interval are twofold: first, isolated nodes existed in the networks of normal controls when density was lower than 22%. Second, the networks lost their small-world property when density was greater than 52%. We therefore constructed all networks in this density interval to guarantee full connectivity and small-world architecture (particularly for normal controls, as abnormal networks could inherently lose those properties). Thus, the symmetric binary matrix represented the individual functional brain connectivity (Fig. 1d).

### Network attack

We proposed two simple failure models to verify whether hub-attack can lead to AD-like degeneration in brain networks or not. Four topological metrics, i.e. small worldness, clustering coefficient, global efficiency, and characteristic path length (for detailed definitions of these topological properties, see Supplementary materials), were used to evaluate the performance of a network. The first was a hub-attack model, in which we successively removed the nodes and the edges linked with hubs in descending order of nodal degree. The second was a random-attack model, in which we removed the nodes one by one and the edges linking them, in random order. After removal of each node, we re-calculated the topological metrics of the remaining subnetwork. The attack ended when all nodes had been removed.

### Evolution rules

To simulate the rearrangement of connections in functional brain networks from healthy individuals to AD individuals, we proposed an evolutionary model to imitate this pathological process.

Naturally, the starting point of the simulation was the NC group. That is, the initial simulation networks were those of the NC group. The subsequent re-connection and disconnection of edges started from these initial simulation networks. In each simulation step, the probability that a new edge was established and an existing, or old, edge was disconnected were the same. These two basic evolution procedures occurred with the same probability of 0.5 to maintain a relatively fixed network density, or wiring cost. The specific rules by which an old edge was selected for disconnection and a new edge was established, are as follows:

### Edge Disconnection Procedure

As described in the *Introduction*, the AD brain is most likely to suffer lesions in hubs. An edge linking two high-nodal-degree nodes was therefore more likely to disconnect in a simulation step. In this context, each edge in the network was assigned a disconnection probability of:


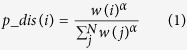


Where *p*_*dis*(*i*) is the disconnection probability of an edge *i*, *N* is the total number of edges in a network, *w*(*i*) is the weight of the edge *i*, and its value is the sum of the two nodal degrees that edge *i* connects. In addition, each edge weight was strengthened via a constant exponential factor, *α*. Thus, an edge connecting hub-like nodes was more likely to disconnect in a simulation step.

### Edge Formation Procedure

The functional brain network in AD becomes randomized along with the pathological process. We therefore established a new edge to link two random nodes to imitate this random process.

**Figure 2:**
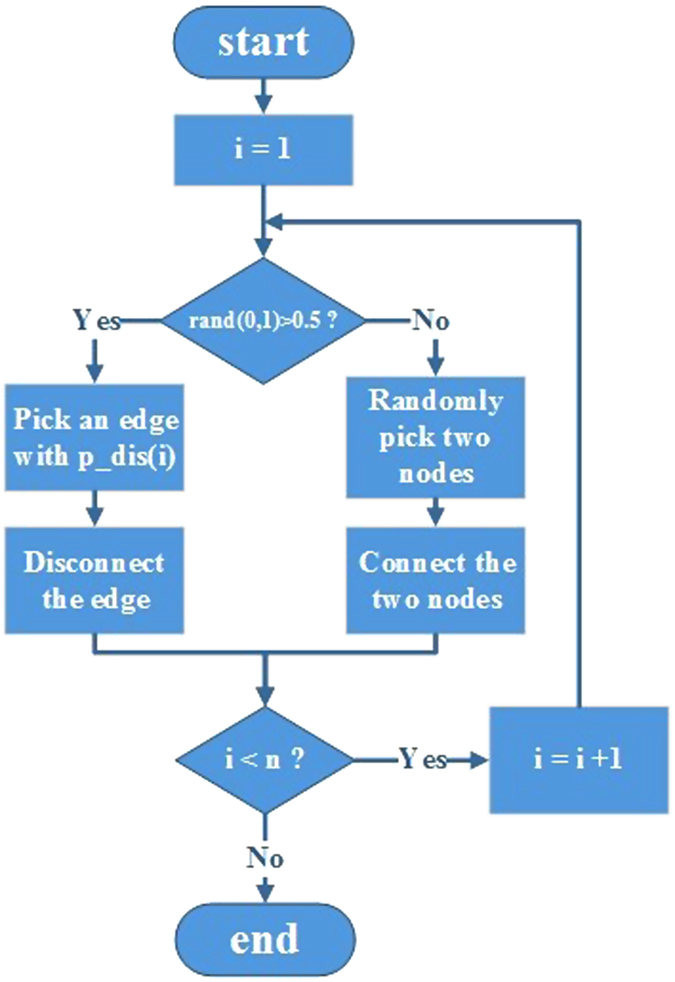
The pipeline of the simulation.

Another issue was how many steps did it require to finish the simulation? It would be improper to end the simulation at the same number of steps for all densities, given that a network with a low density is more sensitive to edge reorganization because a few changes can make a substantial difference in its topology, and vice versa. We therefore related the simulation steps of a network to its density as:


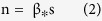


where *n* is the simulation steps of a network, *s* is its density, and *β* is a constant. Consequently, a network with a high density requires more steps to simulate the evolution from the NC group to the AD group, and vice versa. The pipeline of the evolution simulation is shown in Fig. 2.

**Figure 3:**
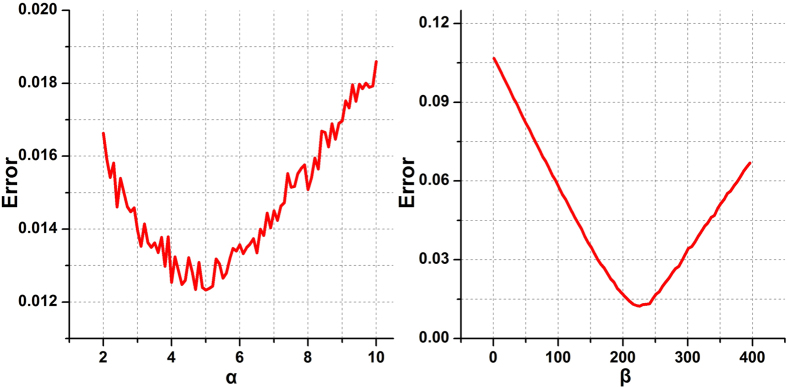
The relationship between the error (cost) and parameters. The left panel shows the relationship between the error and *α* with*β* fixed at 242. The right panel shows the relationship between the error and *β* when *α* was fixed at 5. Both curves have a “valley” shape with minimum values that concur with the DE algorithm.

To obtain the two constant coefficients, *α* and *β*, that best fit the data, we used a differential evolution (DE) algorithm with an error (cost) function as below (Equation 3).


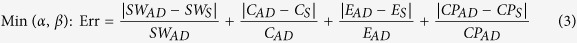


where *SW*, *C*, *E*, and *CP* represent small worldness, clustering coefficient, global efficiency, and characteristic path length of a network respectively. The subscripts *AD* and *S* represent the networks of the AD group and simulation group, respectively. Through the greedy DE algorithm, we obtained the optimal *α* and *β* to minimize the error in topological properties between the networks of the AD group and simulation group. That is, the simulation networks best approached the target AD group with these two optimal coefficients. The DE algorithm was applied to the primary cohort, for which we obtained *α* = 5, *β* = 242. To test the validity of the coefficients, we plotted the error when changing *α* if *β* was fixed at 242, and similarly, changing *β* when *α* was fixed at 5. As shown in Fig. 3, both functions exhibited a ‘valley’ shape, with minima that concur with the DE algorithm. These issues are addressed further in the *Discussion*. The results obtained from the independent cohorts were equivalent. We therefore applied these two coefficients (*α* = 5, *β* = 242) in the evolution experiments for all the three cohorts (one primary and two independent cohorts).

### Random evolution model

We used a random-evolution model as a null model for comparison. The occurrence of connection and disconnection procedures was random. Specifically, which old edge to disconnect and where a new edge was to be established was entirely random. This null model also maintained a relatively fixed wiring cost, although alterations of the topological properties of this model are meaningless, or at least, they cannot be considered as a practical pathological process of AD.

## Results

### Topological differences between NC and AD groups

**Figure 4:**
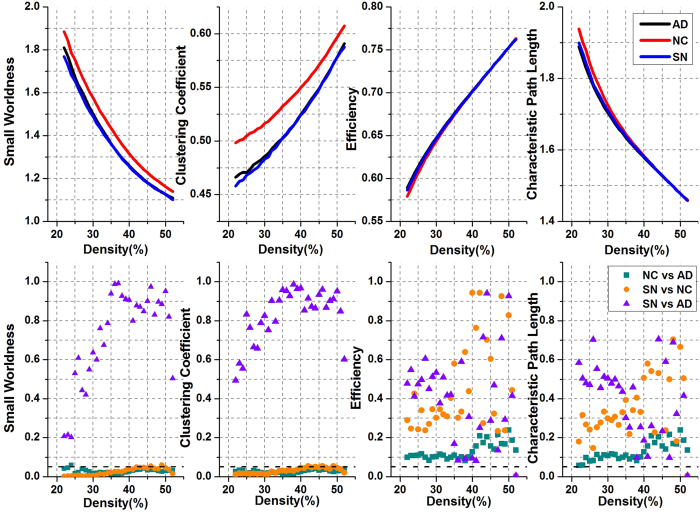
Topological properties and their P-values in the evolution simulation. The first row of the panels shows the topological properties of the AD group (black line), the NC group (red line), and the simulation networks (blue line). The second row of the panels shows the P-values of corresponding properties between the NC and AD groups (cyan square), between the simulation networks and the NC group (orange dot), and between the simulation networks and the AD group (purple triangle).

Four network properties (i.e. small worldness, global efficiency, clustering coefficient, and characteristic path length) were used to evaluate the differences in topological profile between AD and normal controls across the whole density range. As shown in Fig. 4, AD was associated with a significant decrease (*t*-test, P < 0.05) in small worldness and clustering coefficient over the whole density range (except for small worldness for one density). Meanwhile, global efficiency showed a moderate increase at low densities, and converged to the NC group at high densities. Along with global efficiency, there was moderate-to-negligible decrease in characteristic path length. When FDR correction^29^ was implemented for multiple comparisons in each topological property (the correction scale was 31 as there were 31 densities), there was a total of 61 sets of significant difference across the whole density range (Table 2). These results suggest that pathological changes in the brain resulted in topological changes in functional brain networks.

### Network attack results

To illuminate our emphasis on the disconnection between high-degree nodes, we utilized two failure models to validate AD’s damage to network hubs. One is a hub-attack model, the other one a random-attack model.

**Figure 5:**
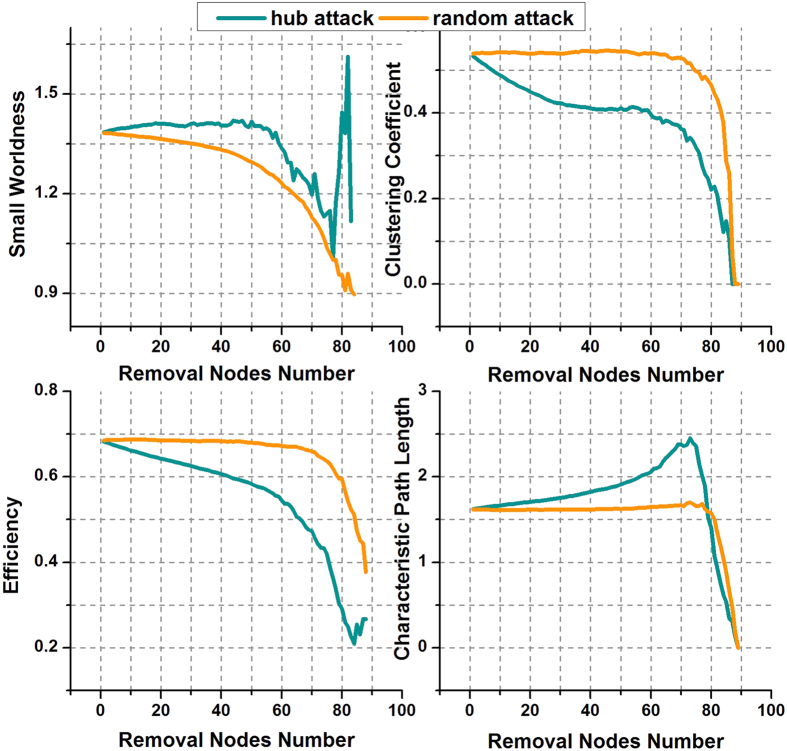
Topological properties of the NC group under hub attack and random attack.

We used these two models to attack the networks of the NC group in the middle of the density range (i.e. 37%). As shown in Fig. 5, all four properties barely changed following random attack. Values were relatively constant before the number of nodes removed reached 70, i.e. 78% of the total nodes. This denotes that healthy brain networks can bear large-scale random failure. That is, if the pathological changes in brain networks in AD are consistent with a random-attack criterion, large-scale damage is required for there to be similarity to actual cases of AD, which are characterized by the various degrees of alteration of topological properties. However, the network properties were sensitive to hub attack. The properties showed rapid decreases when a small quantity of hub-like nodes were removed. This is much more realistic, in that only a small number of important brain regions were affected in the AD cases. These experimental results verify our emphasis on high-degree nodes, or hub-like nodes, in disconnection procedures.

### Simulating the evolution from the NC group to the AD group

The simulation experiment was used to emulate the process of change, or evolution, of the brain networks after incurring AD. The brain networks of the NC group in the primary cohort evolved to the AD group following the rule mentioned in *Section Evolution rules*. As shown in Fig. 4, when the simulation finished, the topological profile of the AD group was well captured by the simulated NC networks (i.e. SN group). That is, the overall performance of the SN group reached the level of the real AD group in terms of all four network properties. Specifically, there were no significant differences for all four properties between the SN and the AD groups (*t*-test, FDR correction, P > 0.05 for all densities). Given this, the SN and AD groups could be considered as deriving from the same population. Compared to the NC group, there were significant decreases in small worldness and clustering coefficient in the SN group in almost all densities (*t*-test, P < 0.05). After FDR correction, 56 sets of significant differences were identified between the SN and NC groups (Table 2), 55 of which were coincidental in terms of those between AD and NC. Namely, 98% of the significant differences that we identified between SN and NC were correct. Moreover, the overall accuracy (i.e., considering both significant differences and non-significant comparisons) was 89% and the sensitivity was 90%. Some increases in global efficiency and decreases in characteristic path length were observed at low densities; these two properties concurred with those of the NC group at high densities. These results are consistent with the behaviours of the real AD group and further indicate the similarity between the SN and AD groups.

**Figure 6:**
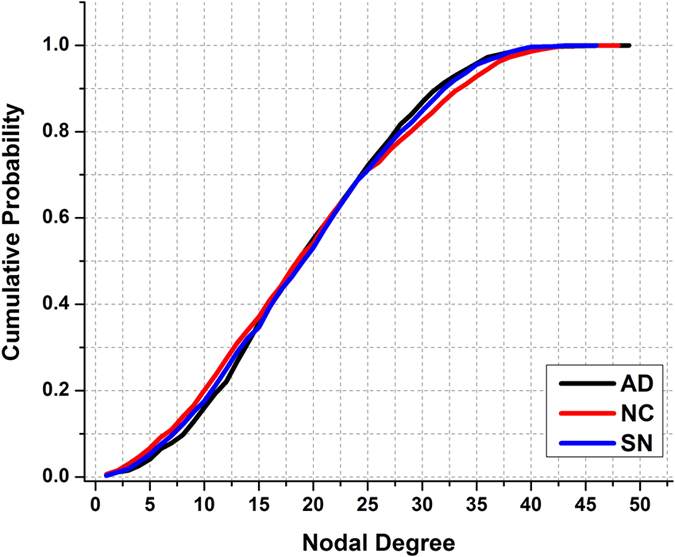
Cumulative distribution of nodal degree in the AD, NC, and SN groups.

Further, we calculated the nodal-degree distribution of the networks in the middle of density range (i.e. 37%) for the NC, AD, and SN groups. As shown in Fig. 6, there was a distinct difference in the distribution between the NC and AD groups. After simulating the evolution, the nodal degree distribution of the SN group approached closely that of the AD group from the initial NC group. Regarding these simulation results, the SN group can be considered a substitute for the real AD group: The simulation started from the NC group, imitated the pathologic changes, and concluded with the SN group being extremely similar to the AD group. We therefore conclude that our simulation model emulated the pathologic processes of AD, in terms of network topology.

### Comparison with random evolution

**Figure 7:**
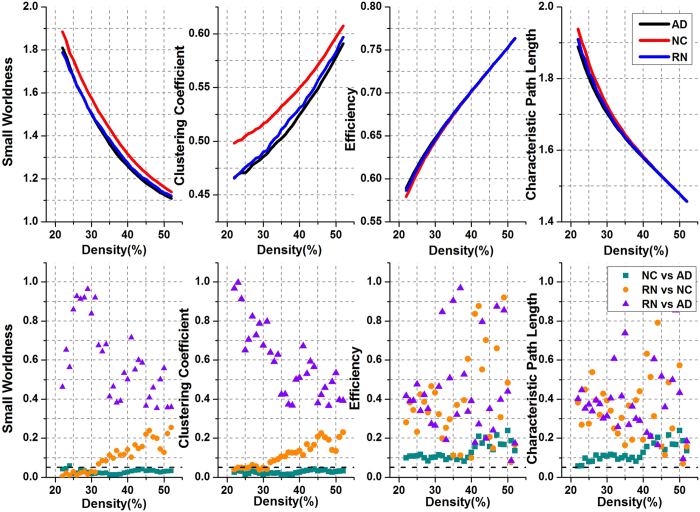
Topological properties and their P-values for random evolution. The first row of the panels shows the topological properties of the AD (black line), NC (red line), and RN (blue line) groups. The second row of the panels shows the P-values of corresponding properties between the NC and AD groups (cyan square), between the RN and NC groups (orange dot), and between the RN and AD groups (purple triangle).

We subsequently simulated random evolution according to the rules in *Section Random evolution model* for comparison. As shown in Fig. 7, networks that evolved randomly from the NC group (i.e. RN group) could not fully capture the performance of the real AD group. Although no significant differences were found between the RN and AD groups, differences between the RN and NC groups were also absent at most (all, if FDR correction was implemented; Table 2) densities. In particular, for densities greater than 32%, none of the four properties exhibited significant differences between the RN and NC groups.

When compared to the NC group, the SN group derived from our model revealed significant differences for 56 densities, similarly to the real AD group. However, the RN group derived from the random-evolution model did not showed any significant difference.

The above experimental results indicate that the random-evolution model could not properly approach the real AD group in terms of topological properties. Our model captured network topology in contrast to the random evolution model, thus demonstrating the validity of our model.

### Verification with independent cohorts

We performed the same experiments on the two independent cohorts, and obtained equivalent results, which strongly validates our model and conclusions. For more detailed experimental results with respect to the independent cohorts, see Supplementary materials.

## Discussion

### Topological differences between the NC and AD groups

We used graph theoretical measures to characterise the differences between individuals with AD and normal controls. In the AD group, we observed significant decreases in the clustering coefficient and small worldness, and moderate to negligible decreases in efficiency and characteristic path length.

The clustering coefficient is a measure of local efficiency of information transfer^30^. Small worldness measures the balance between local processing and global integration^10^,^24^. A significant decrease in the clustering coefficient in individuals with AD indicates a loss of local efficiency, leading to a disrupted small-world architecture in functional brain networks. Although both AD and NC groups exhibited the small-worldness property, the significant decrease in AD compared to NC implies a degeneration. These findings are consistent with numerous fMRI studies^12^,^13^,^14^,^15^, and also with studies that used other imaging modalities^19^,^31^,^32^.

Global efficiency and characteristic path length reflect the global integration in a high-order network system. A highly efficient organization allows for effective transfer of information^30^,^33^. In our study, the characteristic path length showed a moderate decrease at low densities, and converged to the magnitude of the NC group at high densities. These alterations were verified by global efficiency. Specifically, a slightly higher global efficiency can be seen at low densities, whereas any changes were marginal at high densities. However, there is no consensus with respect to how these two measures differ between NC and AD groups^2^,^34^. Sanz-Arigita *et al.* found a decrease in characteristic path length in AD^15^, whereas and Supekar *et al.* found no changes^13^, consistent with our results. However, Zhao *et al.* found a decline in global efficiency and an increase characteristic path length in an AD group^24^. This discrepancy may be due to the different ways the functional brain networks were constructed. Nevertheless, changes in the four topological properties indicate that the pathological processes occurring in the brain indeed resulted in wiring alterations in functional brain networks.

### Evolution model

The brain is a plastic system. Dynamic and static factors coexist in pathological changes of functional brain networks. We therefore cannot fully extract the continually changing brain via a deterministic connectome pattern. In this study, we proposed a novel non-deterministic model to simulate dynamic pathological processes in functional brain networks, as normal individuals transition to individuals with AD.

Not only connections but also disconnections were considered in our model. Each of these two basic evolutionary procedures represents 50 percent of the total probability to maintain a relatively fixed wiring cost. Some previous studies have suggested that cortical hubs are critical regions in Alzheimer’s disease^16^,^17^,^18^,^19^, and have an increased susceptibility to the effects of brain diseases^35^ due to their central role and high cost^16^. Many shortest topological paths pass through hubs, which are highly connected in the brain network. These hub regions therefore easily suffer from trans-synaptic pathological processes that originate in other regions. Further, hub regions help propagate transneuronal degeneration by virtue of their central role^36^. Hub regions have relatively high metabolic demands, rendering neurons connected to them vulnerable to metabolic stress and activity-dependent degeneration^16^,^35^. In particular, when some regions are recruited for compensation, the activity level may increase beyond their own baseline^35^,^37^. In contrast, the damage to the hubs usually results in rapid network fragmentation^38^, leading to degeneration of brain integration; this may be closely associated with the complex brain dysfunctions experienced by patients with AD. Hence, in the disconnection procedure, our model emphasized failures of edges that linked two hub-like nodes, i.e., an edge linking high nodal-degree nodes had a greater probability of suffering a disconnection, and vice versa. We strengthened each edge weight by a constant exponential factor *α*. As we can see in Fig. 3a, the difference between the simulation and real AD groups rose as *α* deviated from the minimum at *α* = 5. This may be interpreted as when *α* is too small, the importance of hub-like nodes is inadequate. When *α* is too large, almost all disconnections are suffered on hubs, which is unrealistic in real brain networks. As a result, *α* in our study was assigned using a DE algorithm. This disconnection strategy weakens the dominant position of hubs in the brain network, leading to a rise in the cost of information transfer. We infer that this is the reason for the disruption of small-world architecture in AD individuals. In turn, the loss of small worldness in AD reflects a randomised shifting. Therefore, in the connection procedure, we randomly picked two nodes to connect, to imitate such a random process. Accordingly, a normal brain network evolved to an AD-like brain network.

### Model evaluation

We used four topological properties to evaluate our model. After the simulation has finished, the topological profile of the AD group was well captured by the SN group over a large density range. Specifically, the SN group was also characterized by significantly reduced small worldness and clustering coefficient. Although the differences in efficiency and characteristic path length between the NC and AD groups were not significant, with a moderate difference at low densities and no apparent difference at high densities, the SN group still captured the subtle changes over the whole density range. Moreover, no significant differences were identified between the AD and SN groups for any of the four properties, indicating that they derived from the same population. However, the null model could not fully capture the performance of the real AD group, indicating that our specific formulation of the evolutionary model was effective.

We also calculated the nodal degree distribution of the three groups for a density of 37%. As shown in Fig. 6, the distribution of the SN group approximated that of the AD group. Although it is questionable to define a threshold nodal degree value to determine hubs, we considered the nodes with a nodal degree greater than 50 as ‘hub-like’ nodes. We found that the percentage of ‘hub-like’ nodes was 7.2% in the NC group, which was higher than the 4.6% of the AD group. After our model evolution, the percentage of ‘hub-like’ nodes in the SN group was 3.8%, a value that was close to that of the real AD group and lower than that of the NC group. Additionally, we simulated the evolution 100 times, and counted the number of disconnections that each region suffered (see Supplementary materials). Of the ten regions that suffered most, many are closely related to AD, such as the temporal gyrus. Although these observation needs refinement by accurately quantifying what a hub is, which is not the primary goal of this research, we nevertheless found a loss of hub-like nodes in AD individuals and a satisfactory outcome of our model.

The results of the network attack further illuminated our emphasis on disconnection between high-degree nodes. Compared to the random failures, the hub attack generated much more realistic outcomes. The human brain network showed robustness to random failures but fragility under hub attack, consistent with many previous studies, indicating that hubs are possibly vulnerable in AD^16^,^17^,^18^,^19^.

Finally, our experimental results were verified using two independent cohorts, and similar results were obtained, supporting our conclusion that the evolutionary model approximated, in satisfactory manner, dynamic alteration of functional brain networks in AD.

## Conclusion

We proposed a novel evolutionary model to simulate the dynamic pathological alterations of functional brain networks in AD, and tested the model via computational experiments. Four topological metrics were utilized to characterize different groups. Results showed that our model captures the dynamic process of the rearrangement of edges in functional brain networks during pathological changes. This model provides us with a new perspective by which we can investigate the potential mechanisms of AD in functional brain networks.

## Additional Information

**How to cite this article**: Li, W. *et al.* Simulating the Evolution of Functional Brain Networks in Alzheimer’s Disease: Exploring Disease Dynamics from the Perspective of Global Activity. *Sci. Rep.* **6**, 34156; doi: 10.1038/srep34156 (2016).

## Supplementary Material

Supplementary Information

## Figures and Tables

**Table 1:** Demographics of the three cohorts.

Cohort	Group	Sex	Mean age (years)
Primary Cohort	AD	7m/8f	72.54
	NC	6m/9f	76.63
Independent Cohort I	AD	4m/3f	68.87
	NC	3m/4f	74.03
Independent Cohort II	AD	4m/4f	66.03
	NC	4m/4f	65.80

**Table 2:** Number of significant differences (FDR correction) across the whole density range.

Comparison	Small Worldness	Clustering	Efficiency	CharPath	Total Number
AD vs NC	30	31	0	0	61
SN vs NC	29	27	0	0	56
SN vs AD	0	0	0	0	0
RN vs NC	0	0	0	0	0
RN vs AD	0	0	0	0	0
