# Supplementary material for: Simulating the Evolution of Functional Brain Networks in Alzheimer’s Disease: Exploring Disease Dynamics from the Perspective of Global Activity
Source: Sci Rep. 2016 Sep 28;6:34156. doi: 10.1038/srep34156 (PMC5039719; doi:10.1038/srep34156)
Supplement: Supplementary Information [file srep34156-s1.pdf]

Simulating the Evolution of Functional Brain Networks in Alzheimer's Disease: Exploring Disease Dynamics from the Perspective of Global Activity

Wei Li[1,2], Miao Wang[3], Wenzhen Zhu[4], Yuanyuan Qin[4], Yue Huang[5], Xi Chen[1,2,*]

1. School of Automation, Huazhong University of Science and Technology, Wuhan, 430074, P. R. China.

2. Image Processing and Intelligent Control Key Laboratory of Education Ministry of China, Wuhan, 430074, P. R. China.

3. China Ship Development and Design Center, Wuhan, 430064, P. R. China.

4. Department of Radiology, Tongji Hospital, Tongji Medical College, Huazhong University of Science and Technology, Wuhan, 430074, P. R. China.

5. School of Electrical and Electronic Engineering, East China Jiaotong University, Nanchang, 330013, P. R. China.

*Corresponding author E-mail: chenxi@mail.hust.edu.cn, Tel: +8618971142226.

S1. The names of the ROIs in the AAL atlas

1	Precentral_L	31	Cingulum_Ant_L	61	Parietal_Inf_L
2	Precentral_R	32	Cingulum_Ant_R	62	Parietal_Inf_R
3	Frontal_Sup_L	33	Cingulum_Mid_L	63	SupraMarginal_L
4	Frontal_Sup_R	34	Cingulum_Mid_R	64	SupraMarginal_R
5	Frontal_Sup_Orb_L	35	Cingulum_Post_L	65	Angular_L
6	Frontal_Sup_Orb_R	36	Cingulum_Post_R	66	Angular_R
7	Frontal_Mid_L	37	Hippocampus_L	67	Precuneus_L
8	Frontal_Mid_R	38	Hippocampus_R	68	Precuneus_R
9	Frontal_Mid_Orb_L	39	ParaHippocampal_L	69	Paracentral_Lobule_L
10	Frontal_Mid_Orb_R	40	ParaHippocampal_R	70	Paracentral_Lobule_R
11	Frontal_Inf_Oper_L	41	Amygdala_L	71	Caudate_L
12	Frontal_Inf_Oper_R	42	Amygdala_R	72	Caudate_R
13	Frontal_Inf_Tri_L	43	Calcarine_L	73	Putamen_L
14	Frontal_Inf_Tri_R	44	Calcarine_R	74	Putamen_R
15	Frontal_Inf_Orb_L	45	Cuneus_L	75	Pallidum_L
16	Frontal_Inf_Orb_R	46	Cuneus_R	76	Pallidum_R
17	Rolandic_Oper_L	47	Lingual_L	77	Thalamus_L
18	Rolandic_Oper_R	48	Lingual_R	78	Thalamus_R
19	Supp_Motor_Area_L	49	Occipital_Sup_L	79	Heschl_L
20	Supp_Motor_Area_R	50	Occipital_Sup_R	80	Heschl_R
21	Olfactory_L	51	Occipital_Mid_L	81	Temporal_Sup_L
22	Olfactory_R	52	Occipital_Mid_R	82	Temporal_Sup_R
23	Frontal_Sup_Medial_L	53	Occipital_Inf_L	83	Temporal_Pole_Sup_L
24	Frontal_Sup_Medial_R	54	Occipital_Inf_R	84	Temporal_Pole_Sup_R
25	Frontal_Mid_Orb_L	55	Fusiform_L	85	Temporal_Mid_L
26	Frontal_Mid_Orb_R	56	Fusiform_R	86	Temporal_Mid_R

27	Rectus_L	57	Postcentral_L	87	Temporal_Pole_Mid_L
28	Rectus_R	58	Postcentral_R	88	Temporal_Pole_Mid_R
29	Insula_L	59	Parietal_Sup_L	89	Temporal_Inf_L
30	Insula_R	60	Parietal_Sup_R	90	Temporal_Inf_R

S2. The definitions of the topological properties mentioned in the paper

The characteristic path length (L) represents the mean shortest path length of all nodes:

$$\mathrm{L} = \frac{2}{N(N-1)} \sum_{i>j} dis_{i,j}$$

Where the $dis_{i,j}$ represents the shortest path length between node i and node j

The clustering coefficient of node i is defined as:

$$C_i = \frac{n}{k(k-1)/2}$$

Where the k represents the number of neighbors of node i, and the n represents the number of edges among the neighbors of node i. The mean clustering coefficient is defined as the average value of the clustering coefficients of all nodes:

$$\mathrm{C} = \frac{1}{N} \sum C_i$$

For convenience, we call the "mean clustering coefficient" the "clustering coefficient" in our paper.

The small worldness is usually defined as the ratio of normalized mean clustering coefficient and normalized characteristic path length:

$$\mathrm{S} = \frac{C/C_0}{L/L_0}$$

Where the C_0 and L_0 represent the mean clustering coefficient and characteristic path length of a surrogate random network.

The global efficiency of a network is calculated as:

$$\text{Eg} = \frac{1}{N(N-1)} \sum_{i \neq j} \frac{1}{dis_{i,j}}$$

Where the *N* represents the scale of the network, i.e. the number of nodes in the network.

S3. The ten regions that suffered most in the simulation of evolution

AAL ID	Region
57	Postcentral gyrus, left hemisphere
2	Precentral gyrus, right hemisphere
1	Precentral gyrus, left hemisphere
7	Middle frontal gyrus, lateral part, left hemisphere
4	Superior frontal gyrus, dorsolateral part, right hemisphere
8	Middle frontal gyrus, lateral part, right hemisphere
89	Inferior temporal gyrus, left hemisphere
81	Superior temporal gyrus, left hemisphere
18	Rolandic operculum, right hemisphere
29	Insula, left hemisphere

S4. Experimental results in the independent cohort *I*

As shown in Fig S1, the topological differences between the NC and AD groups were consistent with those in the primary cohort. After evolution, the topological profile of the AD group in the independent cohort *I* was well captured by the SN group.

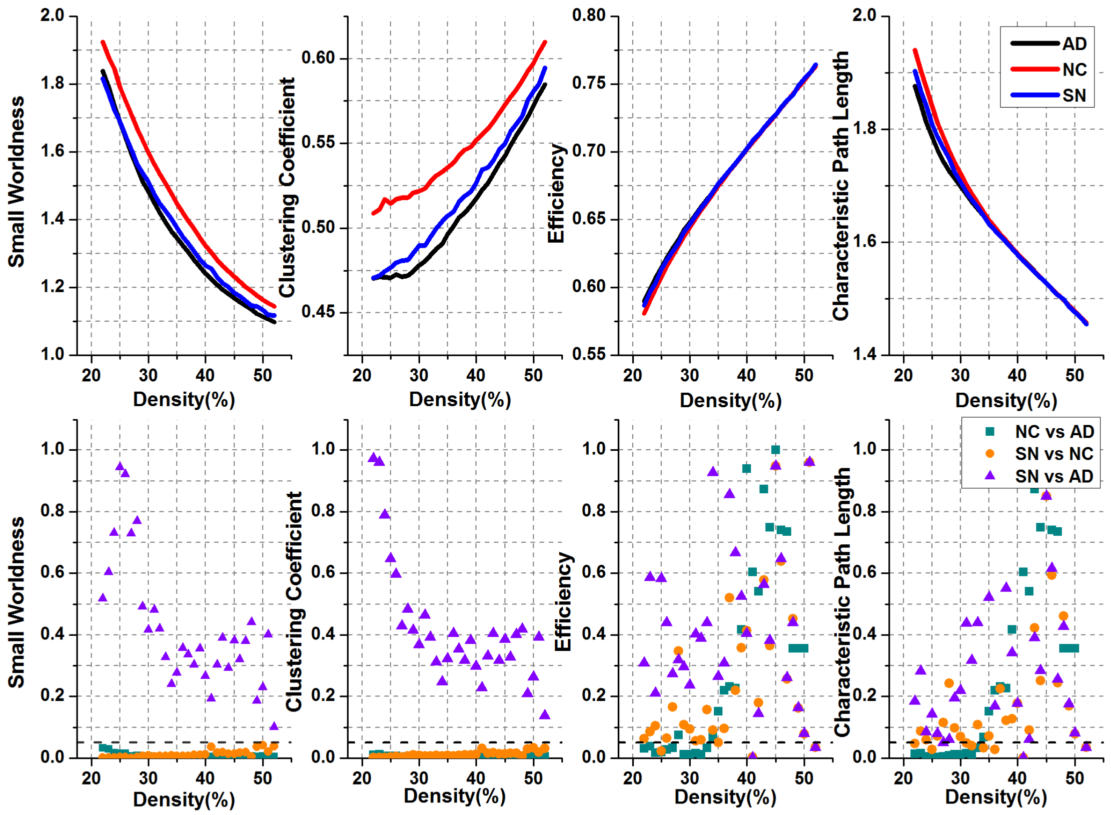

Fig S1

Meanwhile, the nodal degree distribution of the SN group approached closely that of the AD group from the initial NC group (Fig S2).

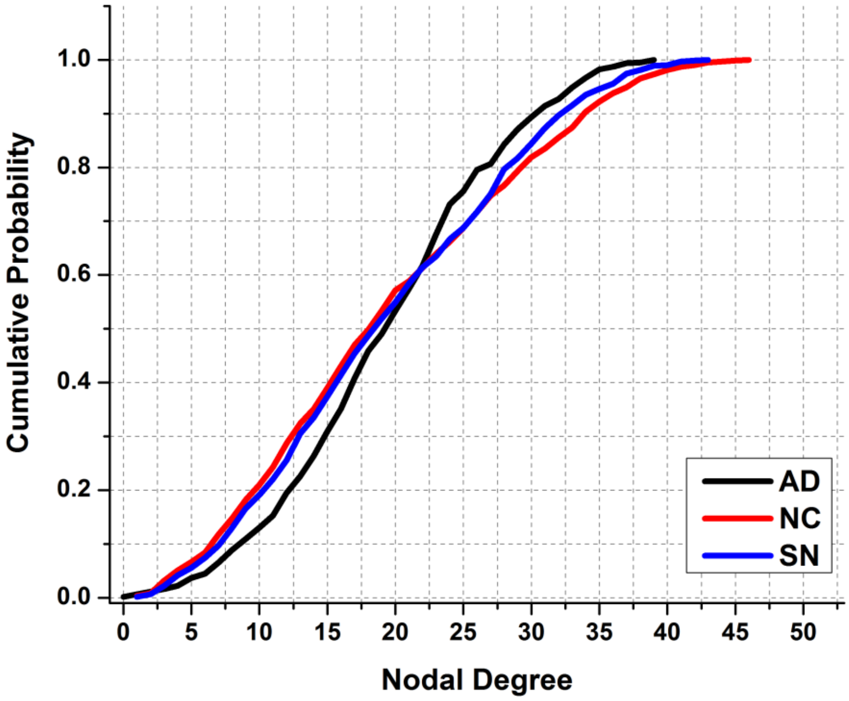

Fig S2

For comparison, after random evolution, the RN group could not fully capture the performance of the real AD group (Fig S3).

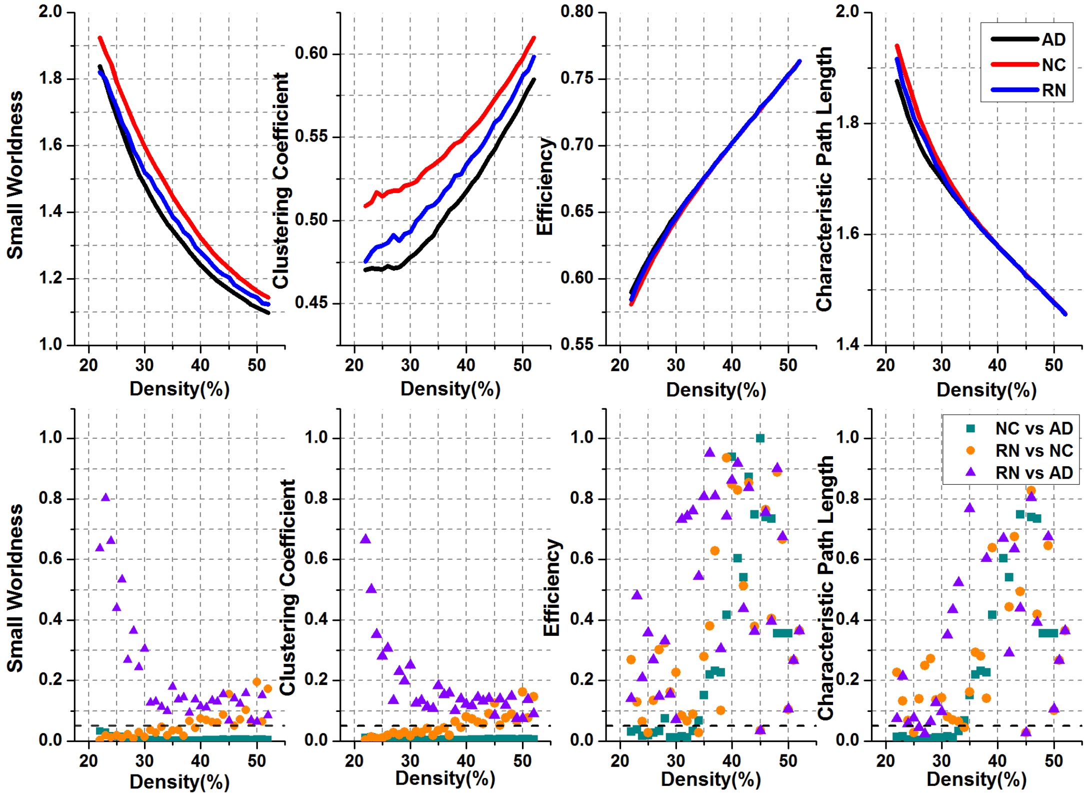

Fig S3

The results of the network attacks are consistent with those in the primary cohort, and support the conclusions in the paper (Fig S4).

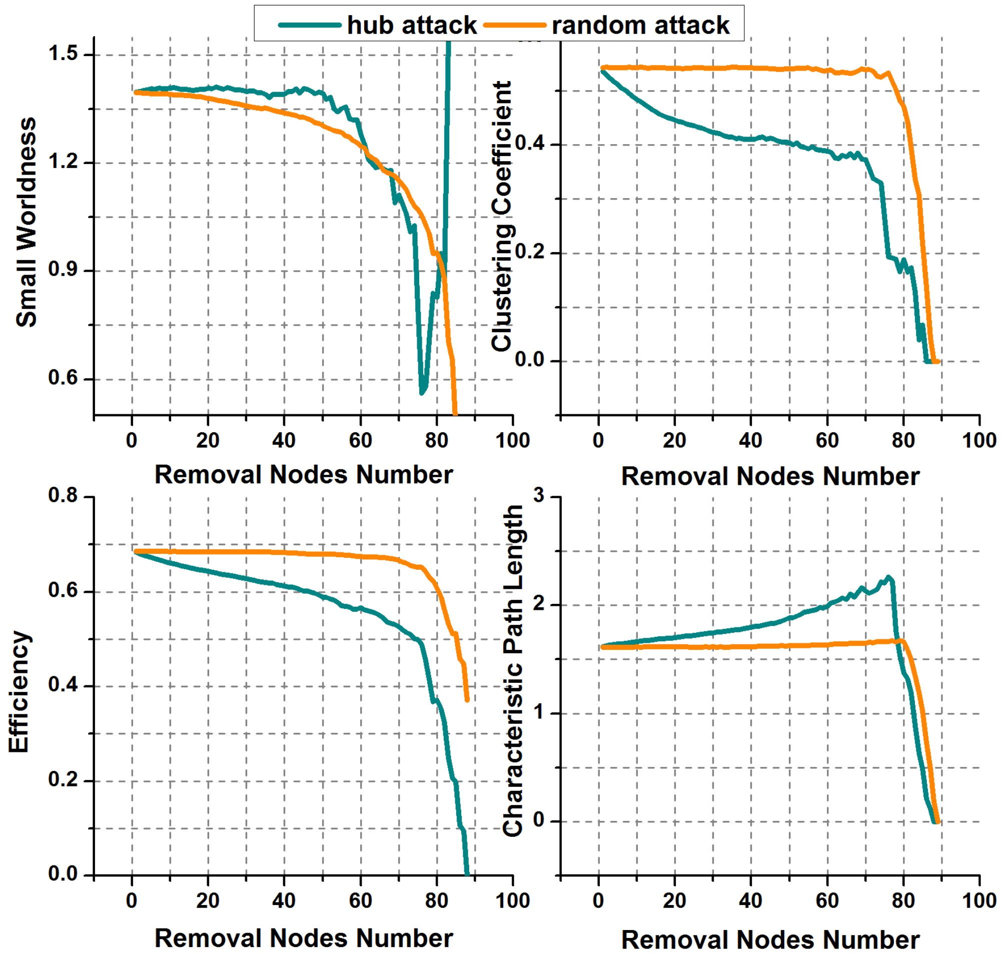

Fig S4

S5. Experimental results in the independent cohort *II*

The results (Fig S5-S8) in the independent cohort *II* were similar to those in the independent cohort *I*, and thus we do not repeat our prior explanations here.

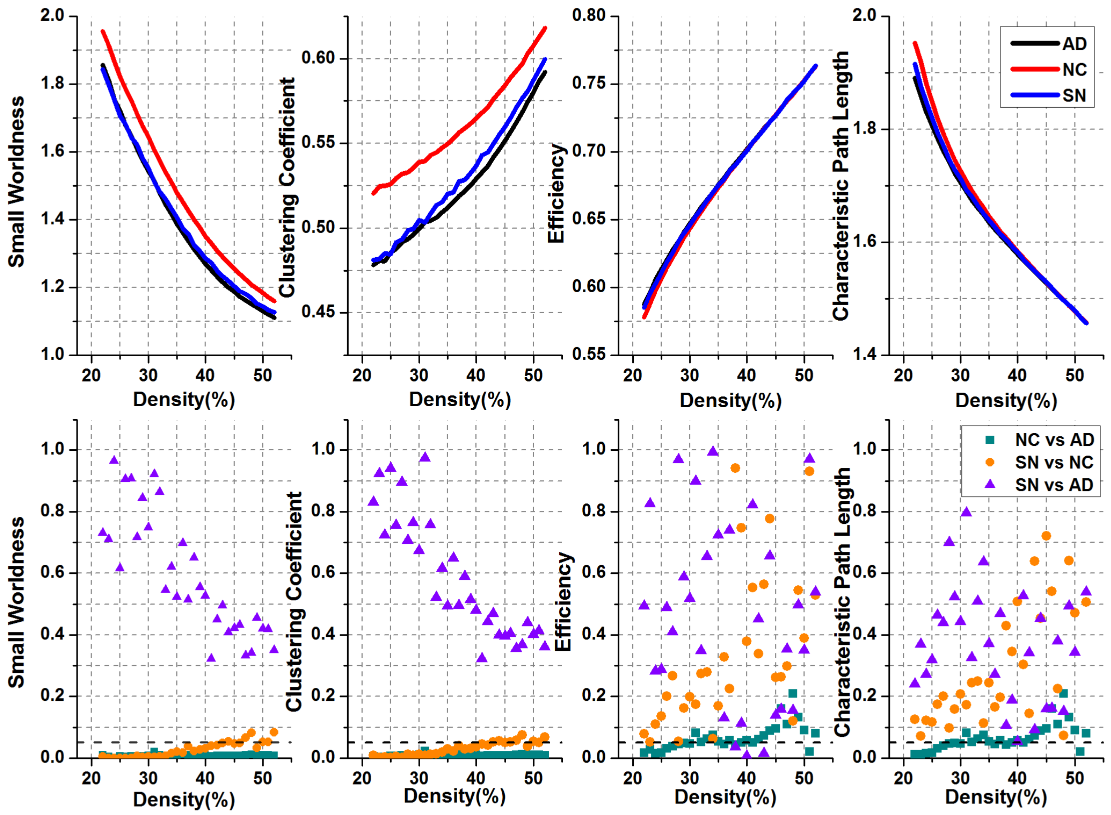

Small Worldness
Clustering Coefficient
Efficiency
Characteristic Path Length
Density(%)
AD
NC
SN
NC vs AD
SN vs NC
SN vs AD

Fig S5

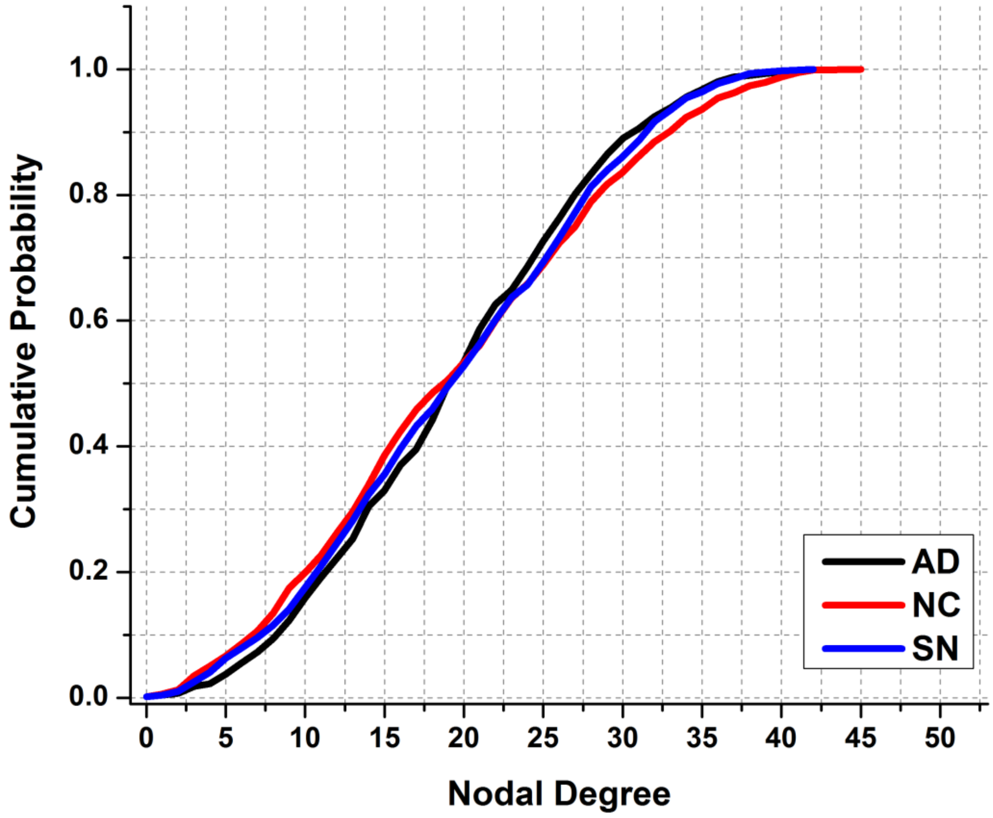

Cumulative Probability
Nodal Degree
AD
NC
SN

Fig S6

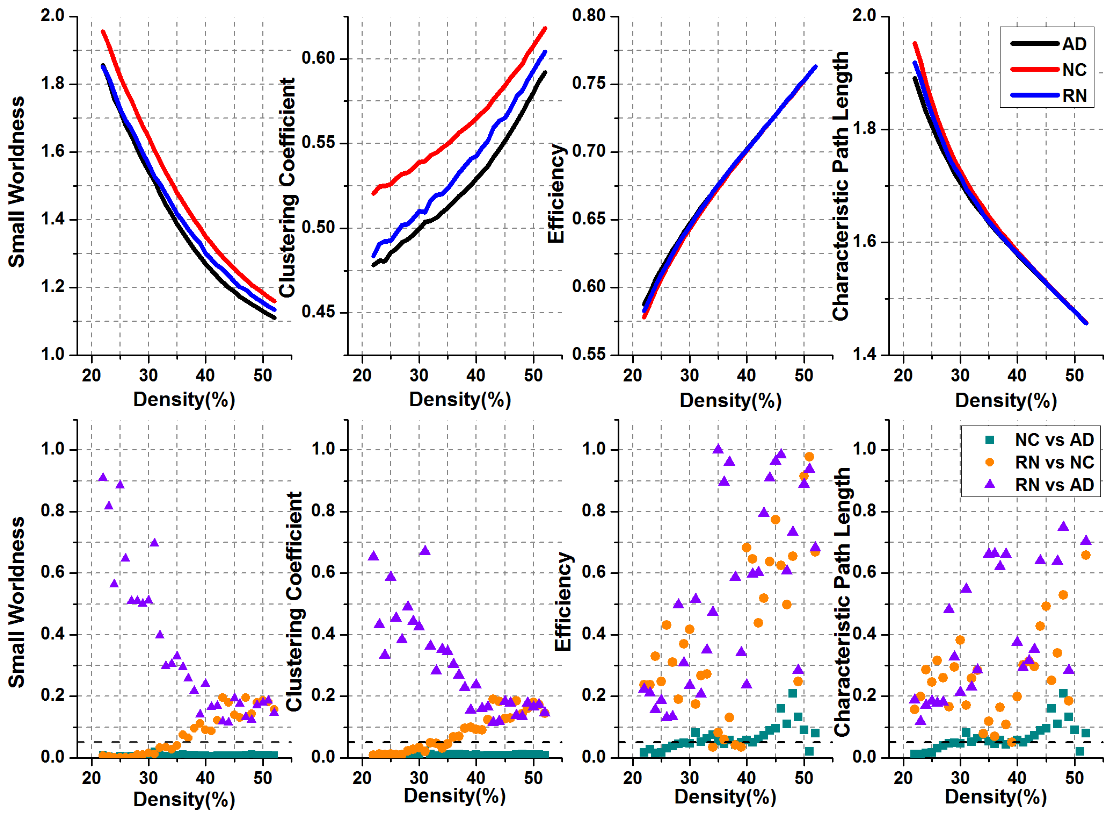

Small Worldness
Clustering Coefficient
Efficiency
Characteristic Path Length
Density(%)
AD
NC
RN
NC vs AD
RN vs NC
RN vs AD

Fig S7

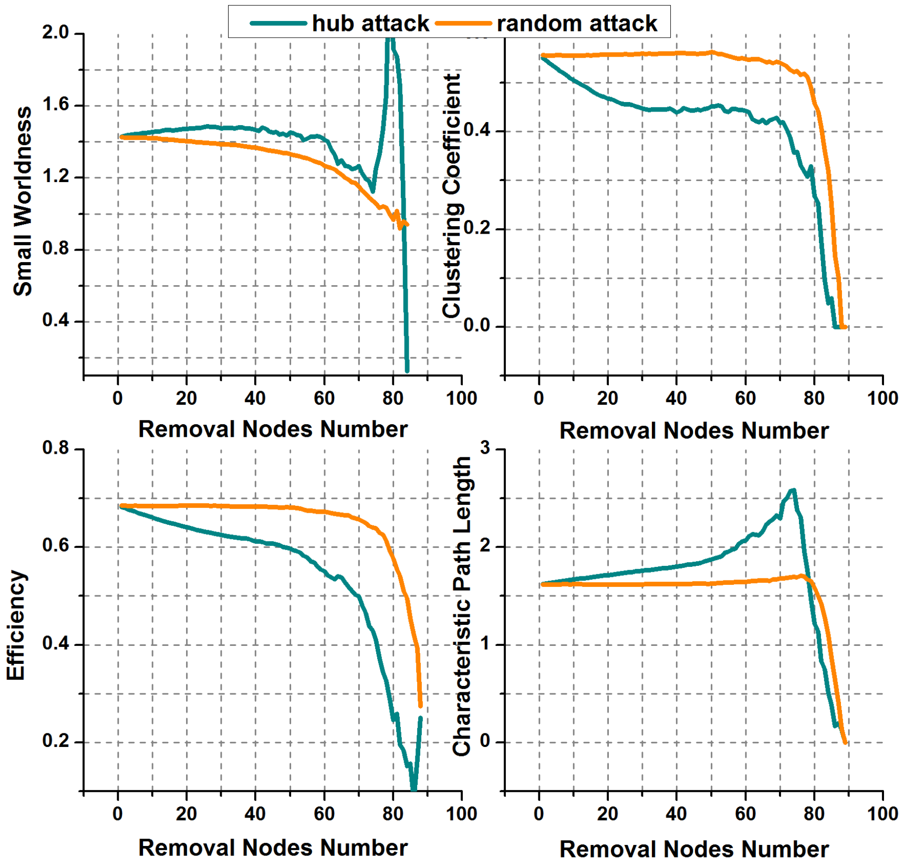

hub attack
random attack
Small Worldness
Clustering Coefficient
Efficiency
Characteristic Path Length
Removal Nodes Number

Fig S8
